# Efficient and selective catalytic hydroxylation of unsaturated plant oils: a novel method for producing anti-pathogens

**DOI:** 10.1186/s13065-021-00748-z

**Published:** 2021-03-29

**Authors:** Ahmed M. Senan, Binru Yin, Yaoyao Zhang, Mustapha M. Nasiru, Yong‐Mei Lyu, Muhammad Umair, Javaid A. Bhat, Sicheng Zhang, Li Liu

**Affiliations:** 1 Glycomics and Glycan Bioengineering Research Center School of Food Science and Technology, Nanjing Agricultural University, Nanjing, 210095 People’s Republic of China; 2 Department of Pharmaceutical Sciences, College of Pharmacy, University of Tennessee Health Science Center, Memphis, TN 38163 USA

**Keywords:** Hydroxy-fatty acids/esters, Functional method, Catalyst, Anti-pathogens, Growth-inhibition

## Abstract

With the increasing demand for antimicrobial agents and the spread of antibiotic resistance in pathogens, the exploitation of plant oils to partly replace antibiotic emerges as an important source of fine chemicals, functional food utility and pharmaceutical industries. This work introduces a novel catalytic method of plant oils hydroxylation by Fe(III) citrate monohydrate (Fe^3+^-cit.)/Na_2_S_2_O_8_ catalyst. Methyl (9*Z*,12*Z*)-octadecadienoate (ML) was selected as an example of vegetable oils hydroxylation to its hydroxy-conjugated derivatives (CHML) in the presence of a new complex of Fe(II)-species. Methyl 9,12-di-hydroxyoctadecanoate **1**, methyl-9-hydroxyoctadecanoate **2** and methyl (10*E*,12*E*)-octadecanoate **3** mixtures is produced under optimized condition with oxygen balloon. The specific hydroxylation activity was lower in the case of using Na_2_S_2_O_8_ alone as a catalyst. A chemical reaction has shown the main process converted of plantoils hydroxylation and (+ 16 Da) of OH- attached at the methyl linoleate (ML-OH). HPLC and MALDI-ToF-mass spectrometry were employed for determining the obtained products. It was found that adding oxidizing agents (Na_2_S_2_O_8_) to Fe^3+^ in the MeCN mixture with H_2_O would generate the new complex of Fe(II)-species, which improves the C-H activation. Hence, the present study demonstrated a new functional method for better usage of vegetable oils.Producing conjugated hydroxy-fatty acids/esters with better antipathogenic properties. CHML used in food industry, It has a potential pathway to food safety and packaging process with good advantages, fundamental to microbial resistance. Lastly, our findings showed that biological monitoring of CHML-minimum inhibitory concentration (MIC) inhibited growth of various gram-positive and gram-negative bacteria in vitro study. The produced CHML profiles were comparable to the corresponding to previousstudies and showed improved the inhibition efficiency over the respective kanamycin derivatives.

**Supplementary Information:**

The online version contains supplementary material available at 10.1186/s13065-021-00748-z.

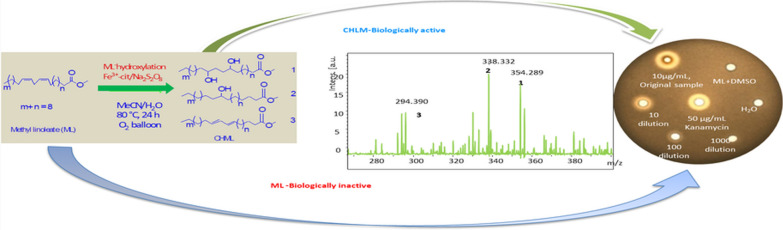

## Introduction

Diverse functions of vegetable oils have attracted their attention for fossil feedstock and industrial applications as renewable biomass to partly replace the fossil resources [1–6]. The hydroxylated plant oils have a potential usage for industrial applications, especially in food industries, due to their lower energy consumption, lesser processing steps. Plants oil conjugates have been used as additives in food, for example; butter, margarine, cooking oils and salad oils, as well as fatty acids supplemental food, biodiesel, paints, greases, and lubricants. However, there have been continued shifts from food to industrial consumption [7–12]. In our previous studies, we reported that the transformation process of fatty acid is based on the reaction of isomerization and/or oxidation to corresponding keto-fatty acids/esters isomers with Pd(II)/Lewis acid catalyst [13, 14]. However, the hydroxy fatty acids containing one or more than one hydroxyl (–OH) groups are remarkable owing to their essential chemical and physical properties. These compounds have diverse industrial and marketing applications, including in food-, cosmetic- and pharmaceutical products [15]. Hydroxy fatty acids also possess suitable applications for paintings, plastics, nylon and carbon source of medicine due to their therapeutic activities [15–18]. For example, 15-hydroxyeicosatetraenoic acid has strong antifungal activities, and it can also be used as an anticancer agent.[19]. Furthermore, hydroxy-methyl linoleate was produced from plant resources by microbial catalyst and hydroxylation of oleic acid with Selenium dioxide-tert-Butyl-hydro peroxide under harsh condition in 72 h [20]. Chang and co-workers reported that the *Pseudomonas aeruginosa* (PR3) had been used as a catalyst for the transformation of unsaturated fatty acids hydroxylation to the corresponding hydroxy fatty acids [21]. Numerous papers have also introduced hydroxy fatty acids from their resources, the production of di-and tri-hydroxy fatty acids (DOD and TOD) combined with low yield in harsh conditions at several days [22–24].

Recently, Tuan et al. reported that castor oil is converted to multi hydroxy-fatty acid by enzyme catalyst [25, 26]. However, the dihydroxy fatty acids were successfully component within no endpoint, and rather harsh reaction conditions, such as high temperature or time of transformation reaction and stoichiometric problems. In previous studies, hydroxy fatty acid such as 7,10-dihydroxy-8-*E*-octadecenoic acid (DOD)was emphatically produced after several days by enzyme catalyst [21–24, 27].

Besides, Persulfate ion S_2_O_8_^2−^ has a much higher radical quantum yield than other oxidant ligands expected of the O_3_. It is also an attractive alternative specialized oxidizing agent in chemistry, which has the ability to oxidize the other substance, such as oxidizing the contaminants in groundwater [28–30]. The activation of sodium persulfate was known by adding the iron Fe (III) as donor of electrons, and the oxidizing target compounds produce a new complex with radicals. However, the reaction mechanism is not well understood [31–33]. In the present study, the iron (III) citrate is significantly activated Na_2_S_2_O_8_ and, it promotes the hydroxylation of methyl linoleate to the corresponding hydroxy-conjugates under simple conditions. Characterization of the hydroxylation system is achieved by using HPLC, MADI-ToF MS, and NMR spectrums. Herein, we propose this a novel catalytic method for preparing the conjugated hydroxyl compounds of plant oils for superior emulsifying, anti pathogens and anti-oxidative agents. The anti pathogenic assays are investigated by using conjugated hydroxy-Linoleic acid methyl ester, especially with minimum inhibitory concentration (MIC) of CHML. This novel strategy designed for an extension food safety and offer potential ways to replace petroleum oil for packaging processes and technologies with very good economical accounts.

## Experimental

### Chemical materials

All reagents were purchased from commercial suppliers and arranged in the laboratory store. (9*Z*,12*Z*)-Octadecadienoic acid methyl ester (ML) and ferric chloride (FeCl_3_)were purchased from (Aladdin Ltd., Shanghai, China). Iron (III) citrate monohydrate (FeC_6_H_5_O_7_·H_2_O) was purchased from (Nanjing chemical reagent Co. LTD). Iron (III) chloride hexahydrate (FeCl_3_·6H_2_O), iron sulfate [Fe_2_(SO_4_)_3_] and ferrous chloride tetrahydrate (FeCl_2_·4H_2_O) were purchased from (Nanjing Chemlin Chemical Co., Ltd.).Iron (II) phthalocyanine (FePC) was purchased from (Aladdin Ltd., Shanghai, China). The sodium cyanoborohydride (NaCNBH_3_), sodium metavanadate (NaO_3_V) and sodium selenite (Na_2_SeO_3_) were purchased from (Sigma-Aldrich Co. LLC). Sodium thiosulfate (Na_2_S_2_O_3_·5H_2_O) and sodium peroxydisulfate (Na_2_S_2_O_8_) were supplied by (Nanjing Lattice, China). Scandium (III) trifluoromethanesulfonate Sc(OTf)_3_was purchased from (Accela Chembio Co., Ltd., Shanghai, China). Dimethyl sulfoxide (DMSO), N,N-dimethylformamide (DMF), acetonitrile (CH_3_CN), tetrahydrofuran(THF), toluene, methanol (MeOH) and dichloromethane (DCM)were all bought from (Sino pharm Chemical Reagent Co., Ltd., Shanghai, China). Methanol used for HPLC purchased from Merck (Nanjing, China).All the media were purchased from Hai Bo Ltd. (Shandong, China). Regular halo test assays were performed for this purpose. Nuclear magnetic resonance (NMR) was performed on an AV400 MHz instrument (Bruker, Beijing, China). Matrix-assisted laser desorption/ionization time-of-flight mass spectrometry (MALDI-ToF–MS) is A Bruker Autoflex Speed mass spectrometer (equipped with a 1000 Hz Smart beam-II laser). High-performance liquids chromatography (HPLC–UV) analysis was carried out in the LCMS 8040 system (Shimadzu Corporation, Kyoto, Japan), all chromatograms were made by Lab-Solutions software. The HPLC analyses equipment with an ultraviolet detector (UV) set at 254 nm.

### General Procedures for catalytic hydroxylation of methyl linoleate (ML) to its derivatives by FeC_6_H_5_O_7_·H_2_O/Na_2_S_2_O_8_ catalyst

In a typical procedure, FeC_6_H_5_O_7_·H_2_O (0.05 mmol) 13.1 mg, Na_2_S_2_O_8_ (6 Equiv.) 59.5 mg were dissolved with 5 mL of MeCN/H_2_O (4:1, v/v) in a glass tube, and then Methyl linoleate 1 M (316.6 µL) was added to the above solution. The reaction mixture was magnetically stirred at 80 °C in an oil bath under anO_2_ balloon for 24 h. The solvent was removed under reduced pressure, and un-reacted materials were washed with cold hexane and filtrated by ethyl acetate and methanol (9:1 v/v). After that, a mixture of solvent was removed under reduced pressure. The crude product was subjected to column chromatography in the eluent solvent as a mixture of petroleum ether/ethyl acetate/methanol (8:1:1, v/v/v), affording the (CHML) products in 88.7 ± 3.3% yield. Controlling the experiments by using FeC_6_H_5_O_7_·H_2_O or Na_2_S_2_O_8_ as the catalyst was carried out in parallel.

### General procedures for detection of methyl linoleate (ML) and its conjugated hydroxy-methyl linoleate (CHML)

#### Nuclear magnetic resonance spectroscopy(NMR)

Methyl-9,12-di-hydroxyoctadecanoate **1**, methyl-9-hydroxyoctadecanoate **2** and methyl-(10*E*, 12*E*) octadecanoate **3** were isolated as mixture (CHML) product, which characterized as well as reported by Kuo et al., Tuan et al., and Kim et al.[15, 17, 22–25]

The ^1^H NMR was recorded in CDCl_3_, ^1^H NMR 400 MHz revealed a peak of carbons that contain hydroxyl groups at 4.19 ppm, while the alpha protons of the carbons nearest to carbonyl groups have a peak at 2.25–2.39 ppm. All methylene groups (CH_2_) appear from 1.81 to 1.26 ppm, and the terminal methyl group has a peak at 0.95 ppm, whereas the ester methyl has a peak at 3.67 ppm. Two tertiary protons appeared at (4.16–3.59 ppm, OH-CH-CH_2_) of carbons that contain hydroxyl groups. All methylene groups (CH_2_) appear from 1.81 to 1.26 ppm and the methyl group has a peak at 0.95 ppm. In the case of carboxylic group, the protons methyl ester group (O-CH_3_) disappeared.

#### HPLC–UV profiling of conjugated hydroxy methyl linoleate (CHML)

Reaction products were identified by LC–MS (Agilent) on a Shimadzu Corporation, Kyoto, Japan, consisting of an LC-30AD pump with COSMOSIL column 5C_18_- MS-II 4.6 ID × 250 mm, at room temperature. The flow rate was adjusted to 0.5 mL/min; water (solvent A) and methanol (solvent B) were used as mobile phases (solvent B). The separation of the conjugated hydroxyl methyl linoleate (CHML) was achieved with the ratio of the elution 75% of solvent B.[34–36]

#### MALDI-ToF mass spectrometric analysis in identification of CHML

Prepared samples were diluted 20-fold with deionized water, then analyzed by matrix-assisted laser desorption/ionization time-of-flight mass spectrometry (MALDI-ToF–MS). A Bruker Autoflex Speed mass spectrometer, it was used for analysing the samples using 2,5-dihydroxybenzoic acid as matrix mass spectra, using Bruker Flex analysis software version 3.3 and were annotated manually.

### Bio-activation and detection of CHML

Several commonly occurring food-borne pathogens including, *Staphylococcus aureus* ATCC 6538, *Listeria monocytogenes* ATCC 15313, *Salmonella typhimurium* ATCC 50013 and *E. coli* O157 CICC 21530 were used for testing the antipathogenic activity of the conjugates of hydroxymethyl linoleate samples, all the bacterial samples recovered from the − 80 °C stock through two times of culturing at 37 °C for 18 h. *S. aureus* was incubated in the Baird-Parkermedium, *S.typhimurium* was incubatedon Xylose Lysine Desoxycholate medium, *L. monocytogenes* was incubated on PALCAM medium and the *E. coli* incubated in Violet Red Bile medium. All the media were obtained from HaiBo Ltd. (Shandong, China). Regular halo test assays were prepared similar to the literature protocol [37]. Unlike a single pure compound, the conjugates of hydroxymethyl linoleate CHML samples are a mixture of hydroxy- octadecanoic methyl esters, and after the purification, the yield of the sample may, therefore, vary for each preparation. In order to measure the amount of the conjugated hydroxy octadecanoic methyl ester which, used the assays more correctly, the sample amount (40 nmol/mL) was calculated based on the HPLC peak areas using a commercial octadecane as standard (10 nmol/mL) as an internal standard for comparison.

### Statistical analysis

All experiments were performed in triplicates. The data was given as average with standard error.

## Results and discussion

To explore the activated sodium persulfate-promoted plant oils hydroxylation with Fe (III) citrate catalyst, we first focused on commercially available (9Z, 12*Z*)-octadecannoic methyl ester as a substrate, using simple Fe (III) citrate monohydrate (Fe^3+^-cit.) with sodium persulfate (Na-pers) as a catalyst, and the results are summarized in Table 1. The chemical reaction was carried out in acetonitrile mixture with water at 80 °C in presence of oxygen balloon, offering 95.3 ± 3.2% neither of the CHML mixture product, while neither Fe (III) C_6_H_7_O_8_ nor of sodium persulfate alone is inactive for methyl linoleate hydroxylation. It can be rationalized by the solubility and fact that there is no extra oxidizing source in the reaction mixture to facilitate the formation of the Fe (III)(citrate)-Na moiety. In this case, may not be realized (S_2_O_8_)^2−^ to initialize the [9, 11]-hydrogen shift mechanism, while the Fe(III)/Fe(II) catalytic cycle for the [9, 12]-hydrogen shifts [38].

**Table 1:** The hydroxylation of methyl linoleate (ML) in presence of the iron catalyst with different sulfate metal ions

Entry	Catalyst	Ligands	Conv.%	Yield of CHLM%
1	FeCl_3_	Na_2_S_2_O_8_	52.2	N.D
2	FeCl_3_·6H_2_O	Na_2_S_2_O_8_	67.3	18.6 ± 1.4
3	FeCl_2_·4H_2_O	Na_2_S_2_O_8_	45.5	21.3 ± 5.4
4	Fe_2_(SO_4_)_3_	Na_2_S_2_O_8_	58.1	13.5 ± 4.6
5	FePc	Na_2_S_2_O_8_	> 90	N.D
6^a^	Fe^3+^-cit·H_2_O	Na_2_S_2_O_8_	100	95.3 ± 3.2(88.7 ± 3.3)
7	Fe^3+^-cit·H_2_O	Na_2_S_2_O_3_·5H_2_O	87.9	81.7 ± 3.5
8	Fe^3+^-cit·H_2_O	NaO_3_V	82.0	14.2 ± 4.2
9^b^	Fe^3+^-cit·H_2_O	Na_2_SeO_3_	100	18.3 ± 5.2
10	Fe^3+^-cit·H_2_O	NaCNBH_3_	100	21.3 ± 2.1
11^c^	Fe^3+^cit· H_2_O	Na_2_S_2_O_8_	100	33.4 ± 2.5
12^d^	Fe^3+^-cit·H_2_O	Na_2_S_2_O_8_	97.2 ± 1.3	73.2 ± 5.2
13	Fe^3+^-cit·H_2_O	–	19.7 ± 2.1	11.5 ± 1.5
14	–	Na_2_S_2_O_8_	100	38.2 ± 4.1
15	Fe^3+^-cit·H_2_O	Sc(OTf)_3_	54	N.D
16	Fe^3+^-cit·H_2_O	H_2_O_2_	80.2 ± 5.5	59.4 ± 5.2

Condition: in mixture solvent MeCN/H_2_O (v/v, 4 mL/1 mL), the 1.0 mmol (316.6 µL), of methyl linoleate was added to the solution containing Fe(III) citrate -monohydrate 0.05 mmol and Na_2_S_2_O_8_ (6 equiv.). Reaction mixture was stirred 24 h at 80 °C under O_2_ balloon. Yield determined by HPLC with internal standard

^a^Isolated and, determined yield by ^1^H NMR analysis with internal standard, N.D. = Not detected

^b^Reaction at 50 °C

^c^Reaction under Argon

^d^Reaction under air

Although sodium-persulfate is a strong oxidizing agent, however, if it’s used alone as catalyst, the hydroxylation yield is offered 38.6 ± 4.4% of CHML and carboxylic acid was generated through hydrolysis of the ester.

In control experiment (entry 13), using Fe(III) C_6_H_7_O_8_ lone as a catalyst offering yield 11.5 ± 1.5% of CHML products from (9Z,12Z)-octadecadienoic acid methyl ester (ML) hydroxylation. Adding Na-metal ions would be greatly promote Fe(III)-catalyzed (9Z,12Z)-octadecadienoic acid methyl ester (ML) hydroxylation, and the improvement of hydroxylation system by highly oxidizing strength-dependent on added metal ions. Using FeCl_3_ or FePc as catalysts with the Na_2_S_2_O_8_ does not generate any improvement for methyl linoleate hydroxylation (Table 1, entry 5). However, adding iron-metal ions such as FeCl_3_·6H_2_O, FeCl_2_·4H_2_O or Fe_2_(SO_4_)_3_ substantially promote the catalytic activity of hydroxylation, providing 18.6 ± 1.4%, 21.3 ± 5.4% and 13.5 ± 4.6% of conjugated hydroxy methyl linoleate CHML with a good selectivity entry 4. In particular, adding Na_2_S_2_O_3_·5H_2_O and/orNa_2_S_2_O_8_ can offer 81.7 ± 3.5%entry7and 95.3 ± 3.2% CHML entry 6respectively in 24 h. The results compared with other sodium metal ions such as NaO_3_V, Na_2_SeO_3_, NaCNBH_3_, and the offered yield of 14.2 ± 4.2% entry 8, 18.3 ± 5.2%entry 9 and 21.3 ± 2.1%entry 10respectively were achieved under current conditions. Na_2_S_2_O_8_ is a redox active and widely used as stoichiometric oxidant or co-catalyst in versatile Fe(II)-catalyzed oxidative C-H activations [39–42]. There were several reports of catalytic transformation of vegetable oil to its conjugates by organometallic catalyst in acetonitrile [43–46]. While a Fe-based catalyst of hydroxylation of the unsaturated plants oil was not reported [47, 48]. In Table 1 entries 11 and 12, the Fe(III)/Na_2_S_2_O_8_ catalyst system in MeCN/H_2_O solution just offered 33.4 ± 2.5% under argon and 73 ± 5.2% in the absences of oxygen balloon.

**Scheme1.**
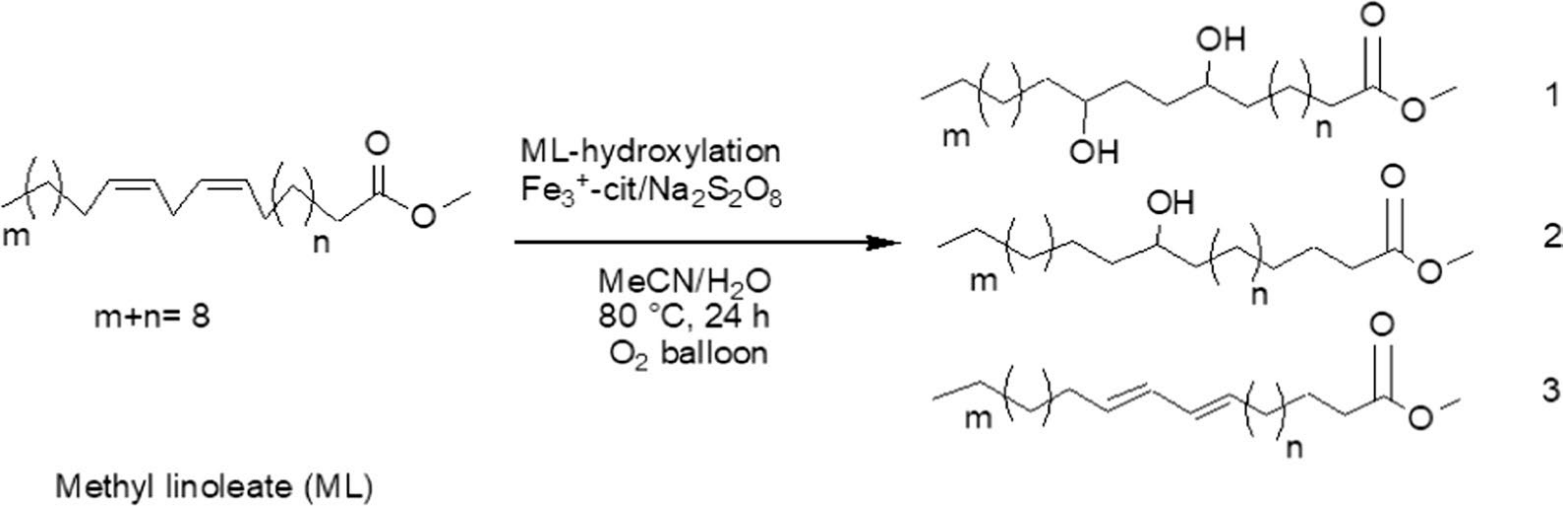
The major products identified by MALDI ToF mass spectroscopy analysis of methyl linoleate hydroxylation with the Fe(III) citrate/Na_2_S_2_O_8_ catalyst

Adding the Na_2_S_2_O_8_ to the mixture reaction sharply accelerated vegetable oils hydroxylation by donor oxygen or accepting of electrons in present water as a nucleophilic attack, it’s best catalytic efficiency for the hydroxylation even better than hydrogen peroxide H_2_O_2_ and the (Sc)^3+^as Lewis acid, Table 1, entries 15, 16 [49–52]. In our case, the produced CHML as a mixture of hydroxy fatty acids from ML was carried out in 24 h with Fe^3+^-cit./Na_2_S_2_O_8_ catalyst as given in a scheme 1

**Fig. 1:**
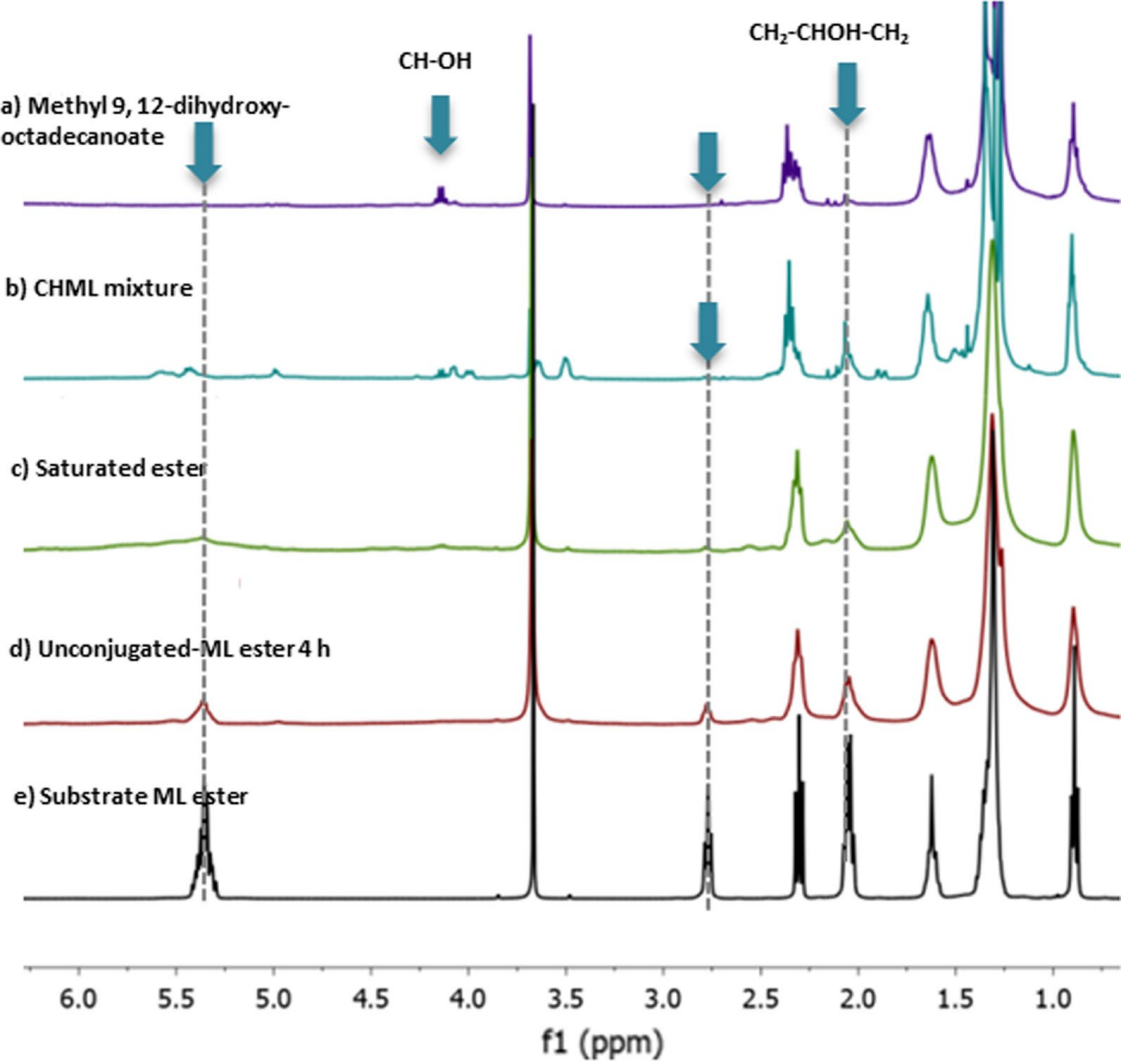
^1^H NMR spectra of methyl linoleate substrate and its conjugated hydroxy methyl linoleate; purified hydroxy methyl linoleate (**a**). Conjugated hydroxy methyl linoleate (CHML) mixture (**b**) and, saturated ester (**c**), unconjugated methyl linoleate isomer 4 h (**d**), and then Methyl linoleate (ML substrate) (e)

In ^1^H NMR characterizations of ML substrate and its conjugated (CHML) products, the chemical shift observed at 2.7 ppm of the methylene protons between the two C = C bonds (CH=CH–CH_2_–CH=CH) in ML, it is shown in Fig. 1e. The chemical shifts of vinylic-hydrogens of the unconjugated methyl linoleate ester appeared at 2.7 and 5.3 ppm in 4 has depicted in Fig. 1d.

Disappearance of the chemical shifts at 2.7 and 5.3 ppm as in saturated ester (Fig. 1c) and, in the case of the new chemical shift was appeared at 3.5–4.2 ppm corresponding to the hydrogens of carbons that contain hydroxyl groups, indicated the hydroxylation product mixture as depicted in Fig. 1b. The isolated product of hydroxy methyl linoleate and its shown in Fig. 1a.The chemical shift for conjugated vinylic hydrogens methyl linoleate disappeared at the peaks at 5.2–5.4, 2.7 and 2.09 ppm, simultaneously, thus excluding the formation of the CHML products. In the case of using Na_2_S_2_O_8_ alone as a catalyst which provided 100% conversion and 38.2 ± 4.1% yield as mixture of conjugated hydroxy methyl linoleate, although the ^1^H NMR spectrum of the isolated products indicated the disappearance of protons of the methyl ester group at 3.6 ppm, thus showing the formation of the linoleic acid as a main product Fig. S1.

The new chemical shift around 3.5–4.2 ppm, simultaneously, disappearance the peaks of vinylic hydrogens and methylene protons at 2.7 and 5.2–5.4 ppm respectively, it’s suggested the hydroxylation reaction occurring on this system [53–55]. Clearly, the roles of addition Na_2_S_2_O_8_ in ML hydroxylation are distinctly different between the presence and absence of the water to the acetonitrile as co-solvent due to increasing the polarity of solvent, the other is to promote Fe^3+^cit-catalyzed hydroxylation of methyl linoleate, addition Na_2_S_2_O_8_ effectively improved the catalytic hydroxylation of methyl linoleate to the desired products under the simple conditions with atmospheric air at 80 °C. In the control experiment, using Fe^3+^-cit/Na_2_S_2_O_8_ as catalyst without water, offered 100% conversion of methyl linoleate. However, CHML products 21.9 ± 4.1% yield were detected in HPLC analysis. The isolated of the main product was identified as saturated ester by ^1^H NMR analysis in Fig. 1c,and Additional file 1: Figures S2, S3, S4, S5 are illustrated the details NMR-spectrum as in supplementary information. The hydrolysis does not happen under current hydroxylation conditions, and ^13^C NMR showed only one carbonyl group as depicted in Figure S6.

Table 2 shows the result of the divers of solvents employed for improving Methyl (9*Z*, 12*Z*)-octadecadienoate (ML) hydroxylation, THF, MeOH, DMSO, and DMF are a poor solvent for this catalysis system. Despite the fact that they got mixed with water are not better than acetonitrile. Adding the water to the reaction mixture significantly supported the catalytic efficiency and Fe^3+^-cit/Na_2_S_2_O_8_-catalyzed methyl linoleate hydroxylation was found with excellent catalytic activity. At the same time, using the acetonitrile alone as a solvent and it is providing only 21.9 ± 4.1% (entry 2) yield of CHML mixture with methyl linoleate isomer. In addition, methanol is a good example ofthe resource of protons donor, which used as a solvent, and the result was also a poor solvent for catalytic ML hydroxylation. Increasing of water ratio in mixture solvent (MeCN/H_2_O) to 2:1 and (1/1, v/v) just obtained 64.3 ± 4.2% (entry 4) and 48.7 ± 2.4% (entry 5) yield of CHML respectively. Increasing of nucleophilic attacks by mixing MeCN/water (4:1, v/v) ratio of solvent and, the hydroxylation provided a better efficiency. The reaction mixture of ML hydroxylation was stirred in the presence of Fe^3+^-cit/Na_2_S_2_O_8_ catalyst for 24 h.

**Table 2:** Fe^3+^-cit/Na_2_S_2_O_8_ catalyzed methyl linoleate hydroxylation to a mixture of conjugated hydroxy-octadecanoate methyl ester in different solvents

Entry	Solvent (v/v)	Conv.%	Yield of CHLM%
1^a^	MeCN/H_2_O (4/1)	100	95.3 ± 3.2(88.7 ± 3.3)
2	MeCN alone	100	21.9 ± 4.1
3	H_2_O alone	98.2 ± 1.8	24.8 ± 2.5
4	MeCN/H_2_O (2/1)	93.7 ± 3.4	64.3 ± 4.2
5	MeCN/H_2_O (1/1)	> 99	48.7 ± 2.4
6	THF/ H_2_O	45.6	ND
7	MeOH	60.4	ND
8	DMSO	45.5	ND
9	DMF	69.7	ND

Condition: In mixture solvent MeCN/H_2_O (v/v, 4 mL/1 mL), the (1.0 mmol) 316.6 µL, of methyl linoleate was added to the solution containing Fe(III) citrate -monohydrate 0.05 mmol (13.1 mg) and Na_2_S_2_O_8_ (6 equiv.). Reaction mixture was stirred 24 h at 80 °C under O_2_ balloon. Yield determined by HPLC with internal standard

^a^Isolated and, determined yield by ^1^H NMR analysis with internal standard, N.D. = Not detected

**Fig. 2:**
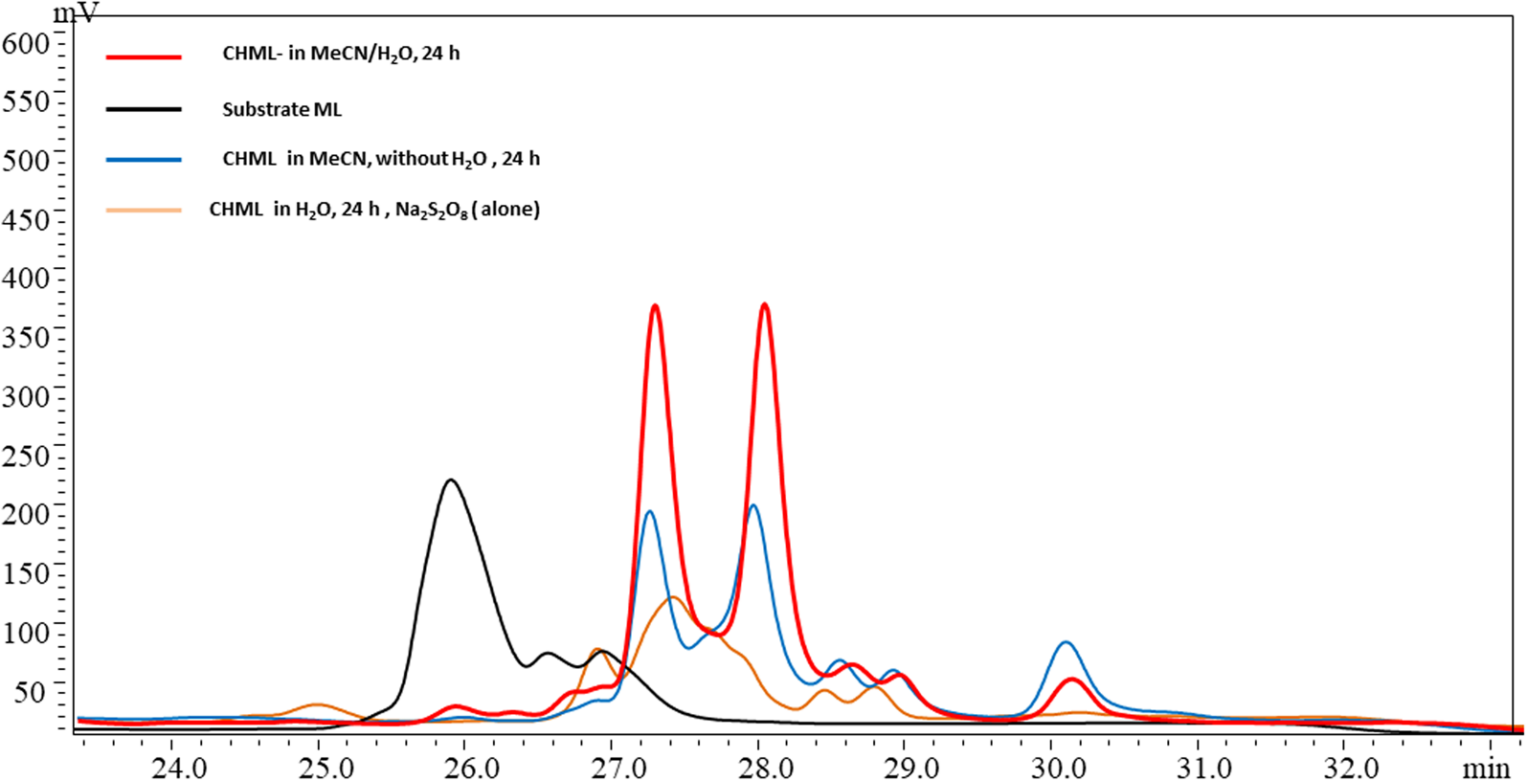
HPLC analysis shows the controlling experiments of ML hydroxylation

The ML conversion was determined by HPLC, which obviously used to separate, identify, and quantify each component in a reaction mixture, as shown in Fig. 2. In spite of adding the water to the reaction solution as co-solvent significantly enhanced ML hydroxylation, the results we found that mono and di hydroxyl octadecanoic methyl ester possess peaks around 27.3 and 28.07 mints respectively. While the negative controls, using Fe(III) alone as catalyst and its offered 19.7 ± 2.1% conversion as shown in Fig. 2 at 27.34 mints. HPLC-separation shows the 100% conversion of methyl linoleate to its conjugated -hydroxy methyl linoleate (CHML), as seen in the red line. The black line shows the peak of the substrate (ML) at rotation time at 26.01 mints. Using the Na_2_S_2_O_8_ as catalyst alone, the peak of the mixture products at 27.34 mints was absorbed on yellowish line with low peaks of CHML ≈38.6 ± 4.4% yield, due to the de-esterification. The grey line shows the products of CHML as a mixture with an excellent yield 95.3 ± 3.2% (Table 2 entry 1).The conversion of ML is calculated at fantail time, [A(ML)^*i*^ and A(ML)^*f*^] the area of ML chromatographic peaks respectively at the initial (zero)and in fantail time.^[10]^A (*St*) is the external standard as show following:

$$C{\text{onversion}}\left( {\text{\% }} \right) = \frac{{\frac{{{\text{A}}\left( {{\text{ML}}} \right)^{i} }}{{{\text{A}}\left( {st} \right)}} - \frac{{{\text{A}}\left( {{\text{ML}}} \right)^{f} }}{{{\text{A}}\left( {st} \right)}}}}{{\frac{{{\text{A}}\left( {{\text{ML}}} \right)^{f} }}{{{\text{A}}\left( {{\text{st}}} \right)}}}}{ } \times { }100$$

where the yields of products (CHML) were calculated at fantail time of reactions, as shown below:

$${\text{Yield}}\left( {\text{\% }} \right) = { }\frac{{\text{mole of CHML}}}{{\text{mole of ML}}}{ } \times 100$$

Alternatively, but the blue line in Fig. 2 showed the lower result of ML-hydroxylation mixture, due to increasing water ratio (2:1, v/v), and that might be caused for hydrolysis of methyl ester. Additional file 1: Figure S3 shows the mixture of products of methyl linoleate hydroxylation 95.3 ± 3.2% yield and, it is worth mentioning that the products mixture of hydroxy-methyl linoleate (CHML) which was further evidenced by MALDI-ToF mass spectrometry. Reading the results of conjugated hydroxy methyl linoleate CHML from Additional file 1: Figure S7, almost no unreacted substrate ML could be detected, and a new mass peaks with an m/z value of 294.39 [(9*E*, 11*E*)-CML, confirmed by ^1^H NMR analysis] [13]. M 330.32, 338.33, and 354.28 were observed, which matches the calculated molecular mass of CHML isomers (294.39 Da for [M]^+^). The product was further verified by detection of a hydroxyl fragment (16.2 Da) by MALDI-ToF MS/MS as shown in Additional file 1: Figure S7.

**Fig. 3:**
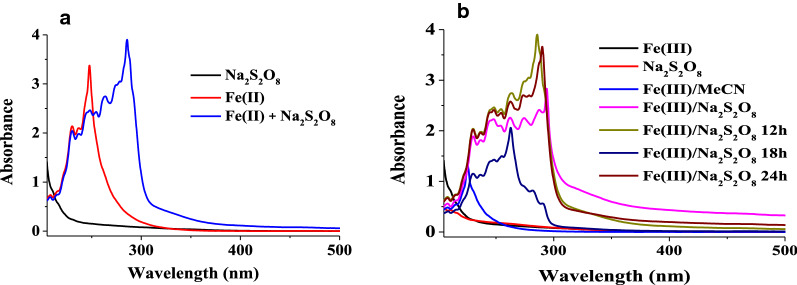
The UV–Vis kinetics of Fe^3+^-cit/Na_2_S_2_O_8_ in 5 mL of MeCN/H_2_O (4:1, v/v) at room temperature

Furthermore, the kinetics of catalyst system obviously determined with UV.vis spectra showed that using the iron alone as catalyst has not band appeared up to 300 nm on beginning reaction’s time, and the band changes to ≈300 nm with adding the Sodium persulfate (Na_2_S_2_O_8_) to Fe^3+^-cit·H_2_O, as we see in blue line at Fig. 3a.

Adding Na_2_S_2_O_8_ to the MeCN/H_2_O solution of Fe(III) citrate at room temperature to order the reaction-kinetic. The absorbance obviously change below 300 nm by adding Na_2_S_2_O_8_ also implicated the formation of new Fe(II) species as well as in acetonitrile alone as blue line in Fig. 3b.The reaction between 2–6 h like in the green-line in Fig. 3b, it can be compared with the characterization results of Fe^3+^-complex which has been studied and published [37, 41].

In particular, adding methyl linoleate to this new species in acetonitrile can immediately trigger the absorbance band maximum around 300 nm, as depicted in Fig. 3b.

**Scheme 2.**
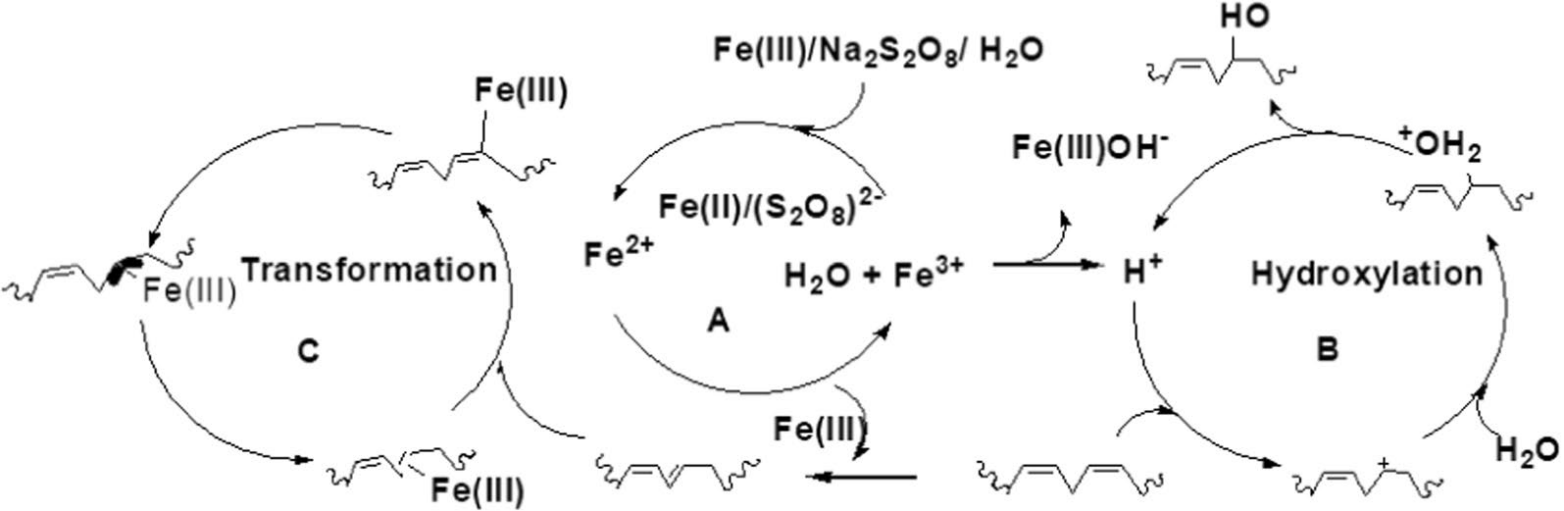
Proposed mechanism for hydroxylation of methyl linoleate to its conjugated hydroxy methyl linoleate by the Fe(III)/Na_2_S_2_O_8_ catalyst

Moreover, the formation of the new complex as a stable species having a characteristic absorbance band at ∼ 300 nm, and the original blue color of the intimidated (Fe^2+^-(S_2_O_8_)^2−^ species which, changes to a pale green of the new species, the new complex formed might be responsible for ML hydroxylation during the time from 12 to 24 h. Adding H_2_O to the reaction mixture facilitate the formation of the Fe(II)-species, formally Fe(II)/Fe(III) cycle was involved in the catalytic cycle (Scheme 2a).

In this catalytic system of internal double bond hydroxylation, it contributed to oxidative/reductive-hydroxylation following [1, 3]-hydrogen shift mechanism, and may not be realized (S_2_O_8_)^2−^ moieties to initialize the [9,11]-hydrogen shift mechanism (Scheme 2b), Fe(II)-species coordinated to C=C bond either from the 9-position or the 12-position, the F(III)/S_2_O_8_^2−^ species next activates the methylene protons which have the chemical shift around 300 nm [13, 38].

Altogether, the conversion was calculated as the consumption of ML and determined by HPLC relative to the initial ML was added, and CHML products are confirmed by matrix-assisted laser desorption/ionization time-of-flight mass spectrometry (MALDI-ToF MS), using HPLC with octadecane as an internal standard. The isolated products were determined by ^1^H NMR relative to the initial ML added with toluene as an internal standard. The selectivity of the total CML was obtained by dividing the average total yield of products (CHML) by the average ML conversion (Scheme 2).

### Growth inhibitions of pathogens by CHML

**Fig. 4:**
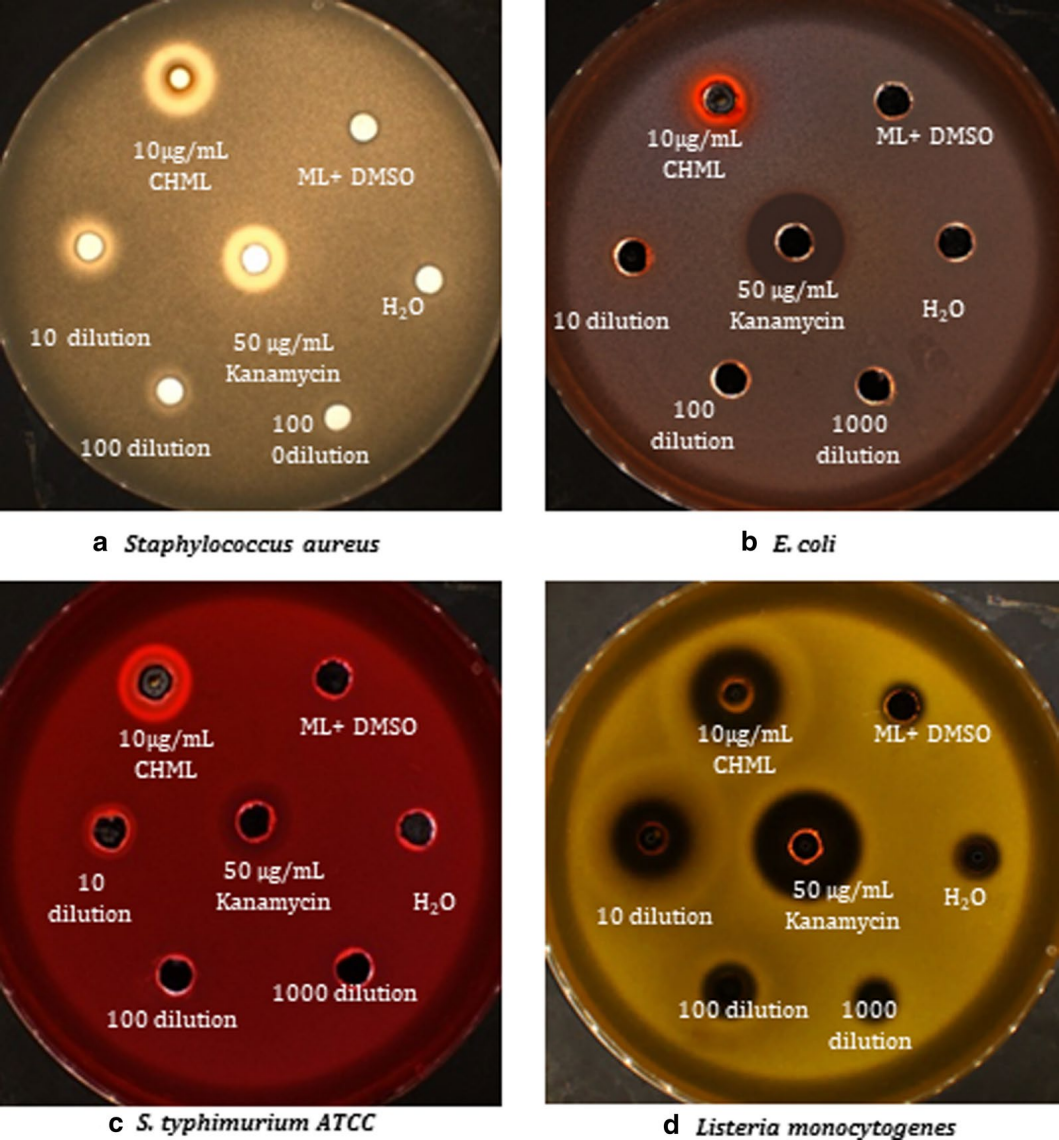
Growth inhibition activity of the mixture of hydroxy- methyl linoleate against: **a** *S. aureus*, **b** *E. coli*, **c** *Salmonella typhimurium*, and **d** Listeria monocytogenes

The results we found in this study may suggest a new method for producing the antimicrobial agents and the spread of antibiotic resistance in pathogens. Methyl linoleate used such a good example on large scale, and the conjugated of its hydroxylation-products can be used as a natural preservative ingredient in pharmaceutical and/or food chemistry. The result of minimum inhibitory concentration (MIC) was tested with the four pathogens; all were more susceptible to hydroxy-methyl linoleate (CHML) than to kanamycin, it is shown in Fig. 4.

The original samples concentrations with highest MIC (10 µg/mL) were chosen and compared with *kanamycin* for the subsequent anti-pathogens activity assay. For the growth inhibition assays no effects from ML (substrate) with DMSO components; similarly, the water showed no activity. Whereas the original samples of CHML were tested and showed a large zone with S. aureus and *Listeria monocytogenes*as depicted in Fig. 4a, d). However, the samples were diluted 10, 100, 1000 times of concentration and dose generate clear zone, and CHML employed with kanamycin as a good comparable, and CHML led to a strong inhibition of growth. The CHML was performed and purified with excellent yield 261.2 mg, 88.7 ± 3.3% of CHML mixture. Four tested strains were selected and used 10 µg/mL of CHML for bacterial inhibition growth in each sample.

The inhibitory potential of methyl linoleate hydroxylated is comparable to the standard clinical dose of kanamycin of 50 mg/mL. In the cases of *E*. coli and *S. typhimurium* simples, the vegetable oils hydroxylated (CHML) showed only moderate growth inhibition as depicted in Fig. 4b, c. Accordingly, this novel functional method was successfully producing the anti-pathogens with an excellent performance.

In this study, hydroxylation of the unsaturated plant oils was investigated by metal catalysts for the first time. Taking advantage of the availability of large amounts of methyl linoleate with a catalyst in our laboratory, we were able to prepare the CHML on large scale, which enables their subsequent use as in antimicrobial assays.

## Conclusions

The hydroxylation of plant oils exhibited the significant role of Na_2_S_2_O_8_ in promoting the Fe(III)-catalyzed methyl linoleate hydroxylation. Adding Na_2_S_2_O_8_ oxidizing to simple iron (III) citrate tri-basic monohydrate as a catalyst can sharply promote its hydroxylation efficiency, even much better than the classic H_2_O_2_ as oxidant legend [11], which highlights the peroxide properties. Persulfate –S_2_O_8_^2−^, also has a high redox potential which, mixing the iron Fe^3+/^Fe^2+^ with persulfate, readily facilitated the generation of new Fe^2+^-species [38, 39, 42]. Noticeably, the hydroxylation was conducted under atmospheric air (oxygen balloon). While previously reported an olefins and or unsaturated fatty acids hydroxylation were generally conducted under harsh conditions, all unlikely Fe^3+^-cit/Na_2_S_2_O_8_ catalyst system demonstrated here.

In addition, these results suggested a new opportunity for improvement the application of vegetable oils methyl ester derivatives in medicals system and food industry. Particularly, it relates to hydroxylated plant oils of superior antibiotic properties. The inhibition growth of different microorganisms with minimum inhibitory concentration (MIC) was investigated by using CHML; the mechanism of growth inhibition mostly attributed to the antioxidative properties of CHML contains hydroxy groups. The conjugated hydroxy methyl linoleate (CHML) as mixture demonstrated a remarkable growth inhibiting gram-positive vs gram-negative bacteria as well as in vitro. Using high performance and analysis, MALDI-ToF mass spectroscopy was employed for determining the CHML. This novel method is suitable for hydroxylating vegetable oils in food industry uses, environment-friendly and future sustainable technology, maintaining the quality of food products, economized field operations, increased the rate and efficiency. Based on these results, we provide recommendations for potential ways in food safety.

## Supplementary Information

**Additional file 1.** ^1^H and ^13^C NMR spectra for conjugated hydroxymethyl linoleate (CHML) and MALID-ToF spectrometry. **Figure S1.** The quantification products by ^1^H NMR Spectrum; Linoleic acid obtained as main product of ML hydroxylation with (Na_2_S_2_O_8_) alone as catalyst. **Figure S2.** ^1^H NMR spectrum of conjugated hydroxy methyl linoleate (isolated product 1). **Figure S3.** ^1^H NMR spectrum of conjugated hydroxy methyl linoleate CHML (Reaction Mixture). **Figure S4.** ^1^H NMR Spectrum of Saturated Ester. **Figure S5.** ^1^H NMR spectrum of original methyl linoleate. **Figure S6.** ^13^C NMR spectrum of isolated product in CDCl_3_ (after removal of the solvent MeCN/H_2_O). **Figure S7.** MALDITOF–mass spectroscopy employed for determining the mixture of conjugated hydroxy methyl linoleate CHML after reaction time 24 h.

## Data Availability

The datasets used and/or analysed during the current study available from the corresponding author on reasonable request. Although, all data generated or analysed during this study are included in this published article and its additional files.

## Abbreviations

Fe^3^^+^-cit.: Iron citrate monohydrate
CHML: Conjugated hydroxy methyl linoleate
ML: Methyl linoleate
HPLC: High performance liquid chromatography
MALDI-TOF: Matrix-assisted laser desorption/ionization time-of-flight mass spectrometry
