# Supplementary material for: Efficient and selective catalytic hydroxylation of unsaturated plant oils: a novel method for producing anti-pathogens
Source: BMC Chem. 2021 Mar 29;15(1):20. doi: 10.1186/s13065-021-00748-z (PMC8008645; doi:10.1186/s13065-021-00748-z)
Supplement: Supplementary file 1 — Additional file 1. 1H and 13C NMR spectra for conjugated hydroxymethyl linoleate (CHML) and MALID-ToF spectrometry. Figure S1. The quantification products by 1H NMR Spectrum; Linoleic acid obtained as main product of ML hydroxylation with (Na2S2O8) alone as catalyst. Figure S2. 1H NMR spectrum of conjugated hydroxy methyl linoleate (isolated product 1). Figure S3. 1H NMR spectrum of conjugated hydroxy methyl linoleate CHML (Reaction Mixture). Figure S4. 1H NMR Spectrum of Saturated Ester. Figure S5. 1H NMR spectrum of original methyl linoleate. Figure S6. 13C NMR spectrum of isolated product in CDCl3 (after removal of the solvent MeCN/H2O). Figure S7. MALDITOF–mass spectroscopy employed for determining the mixture of conjugated hydroxy methyl linoleate CHML after reaction time 24 h. [file 13065_2021_748_MOESM1_ESM.pdf]

Electronic Supplementary Materials

Efficient and selective catalytic hydroxylation of unsaturated plant oils; a novel method for producing anti-pathogens

Ahmed M. Senan,*[1,2] Binru Yin,[1] Yaoyao Zhang,[1] Mustapha M. Nasiru,[1] Yong-Mei Lyu,[1] Muhammad Umair,[1] Javaid Akhter Bhat,[1] Sicheng Zhang,[2] and Li Liu *[1]

Contents:

Figure S1 The quantification products by ^{1}H NMR Spectrum; Linoleic acid obtained as main product of ML hydroxylation with ($Na_2S_2O_8$) alone as catalyst

Figure S2 ^{1}H NMR spectrum of conjugated hydroxy methyl linoleate (isolated product **1**)

Figure S3 ^{1}H NMR spectrum of conjugated hydroxy methyl linoleate CHLM (Reaction Mixture),

Figure S4 ^{1}H NMR Spectrum of Saturated Ester

Figure S5 ^{1}H NMR spectrum of original methyl linoleate

Figure S6 ^{13}C NMR spectrum of isolated product in $CDCl_3$ (after removal of the solvent MeCN/H_2O)

Figure S7. MALDITOF –mass spectroscopy employed for determining the mixture of conjugated hydroxy methyl linoleate CHML after reaction time 24 h

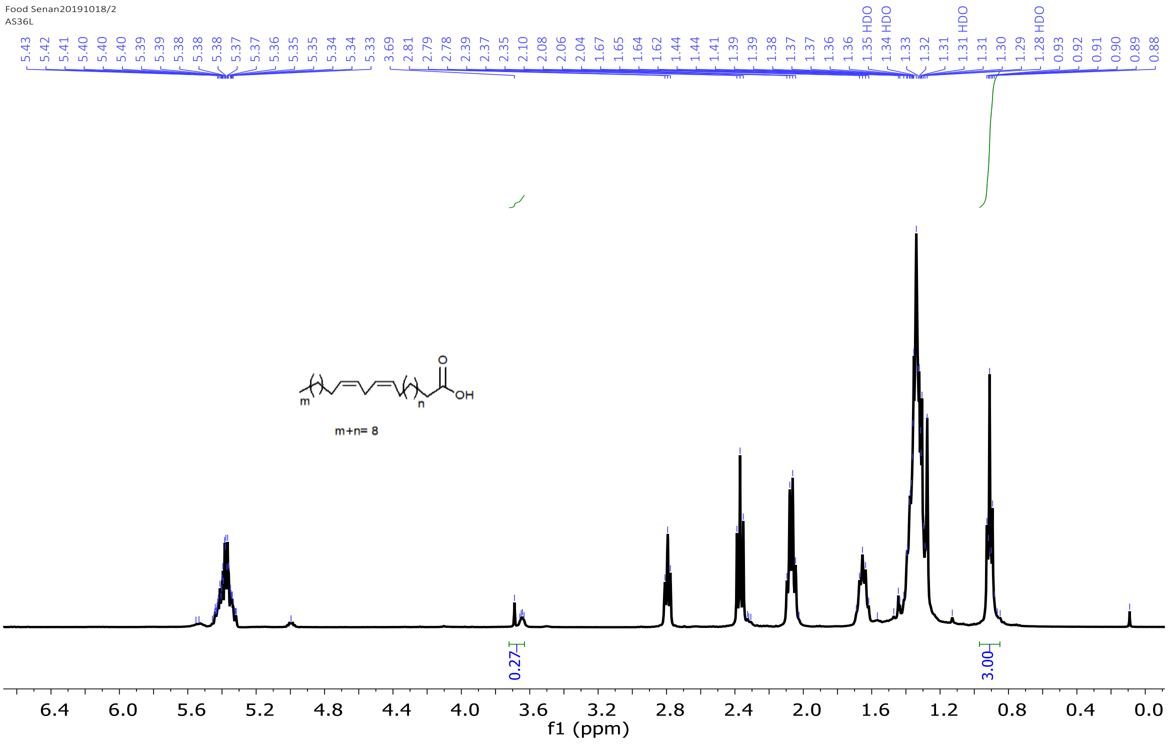

Figure S1 The quantification products by ^{1}H NMR Spectrum; Linoleic acid obtained as main product of ML hydroxylation with ($Na_2S_2O_8$) alone as catalyst

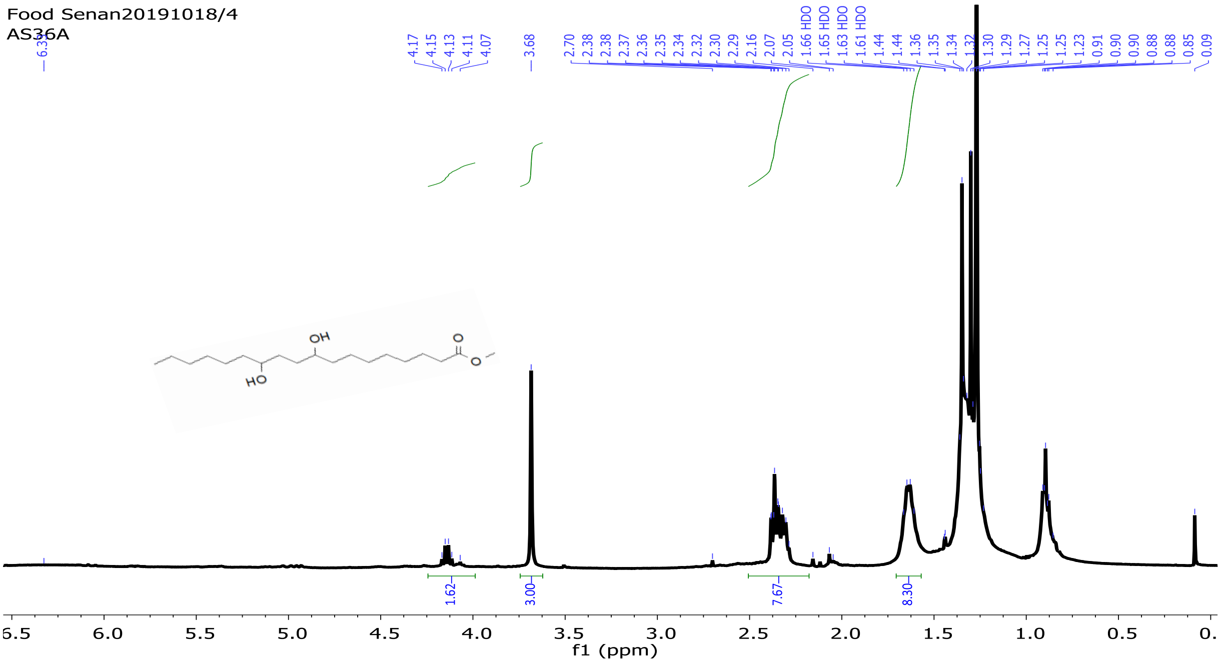

Figure S2 ^{1}H NMR spectrum of conjugated hydroxy methyl linoleate (isolated product **1**)

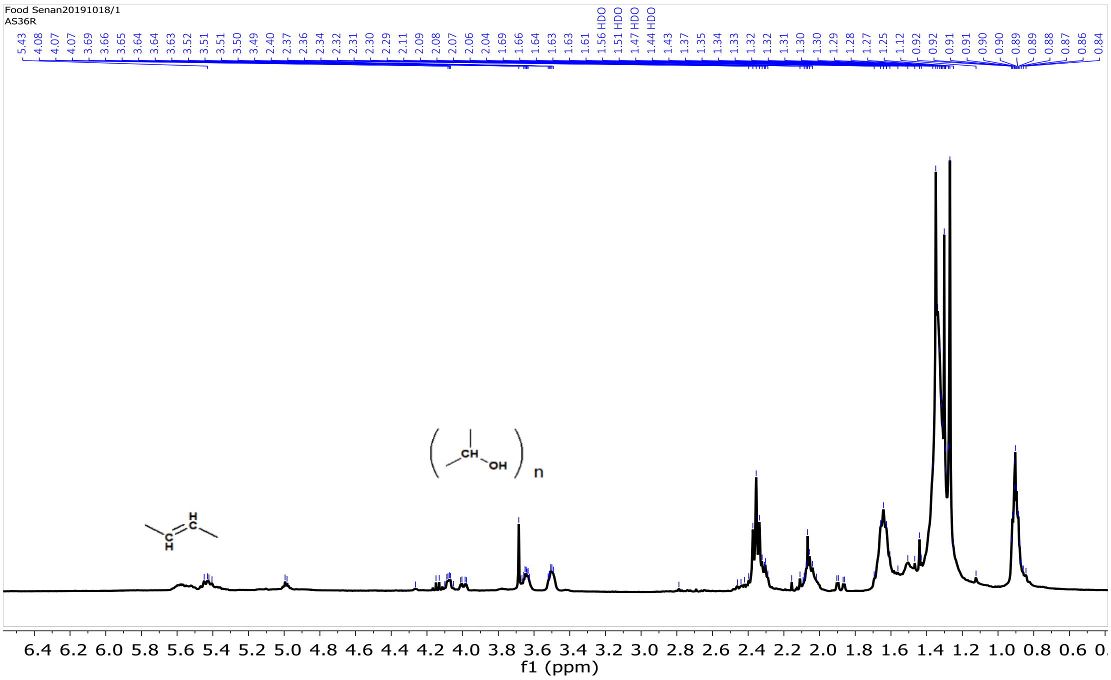

Figure S3 ^{1}H NMR spectrum of conjugated hydroxy methyl linoleate CHLM (Reaction Mixture), n=5

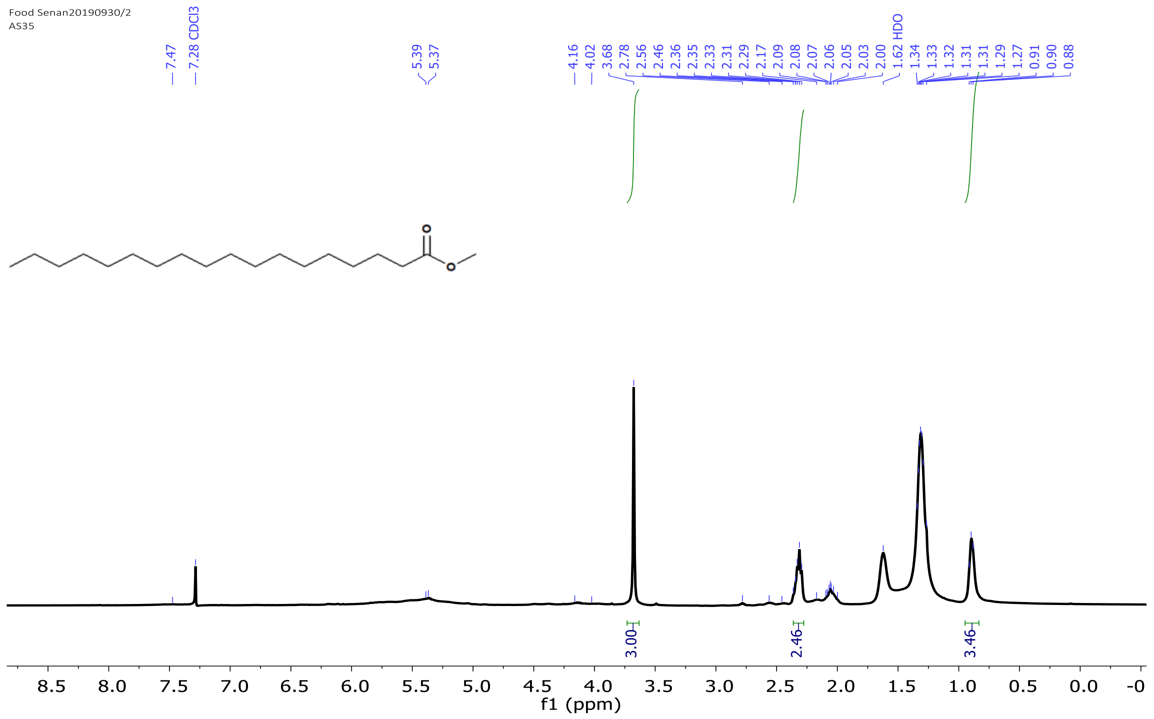

Figure S4 ^{1}H NMR Spectrum of Saturated Ester

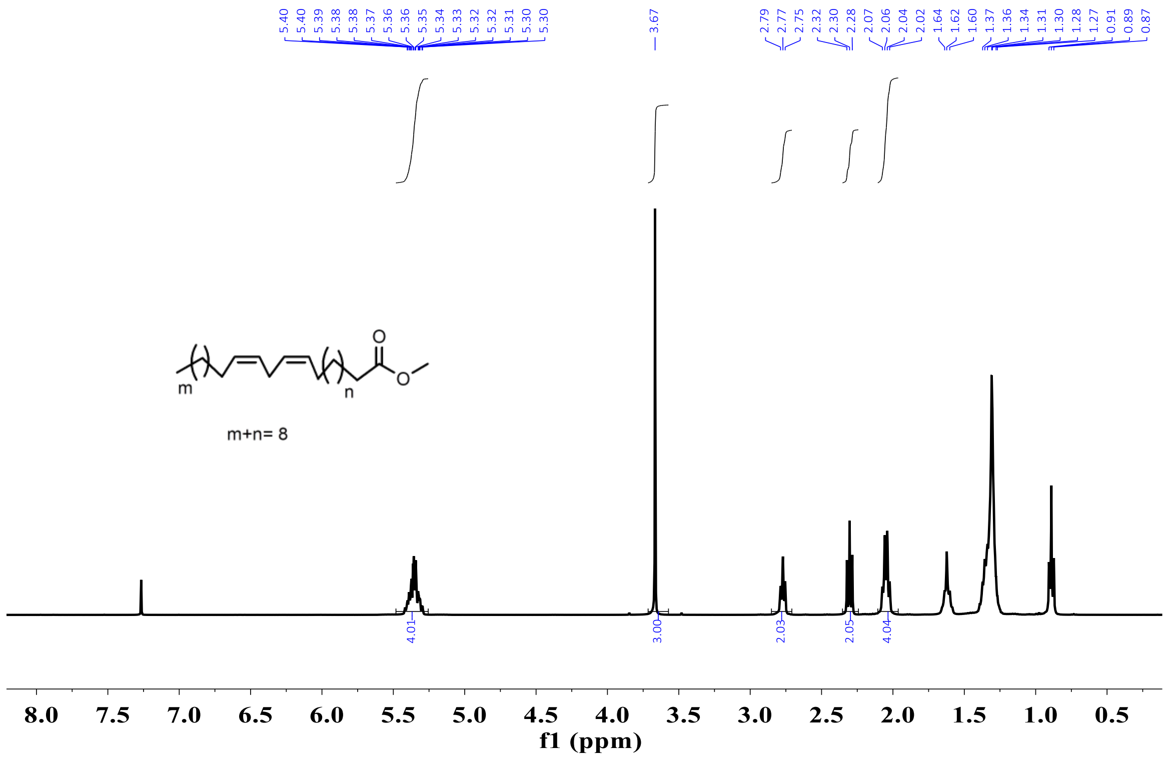

Figure S5 ^{1}H NMR spectrum of original methyl linoleate

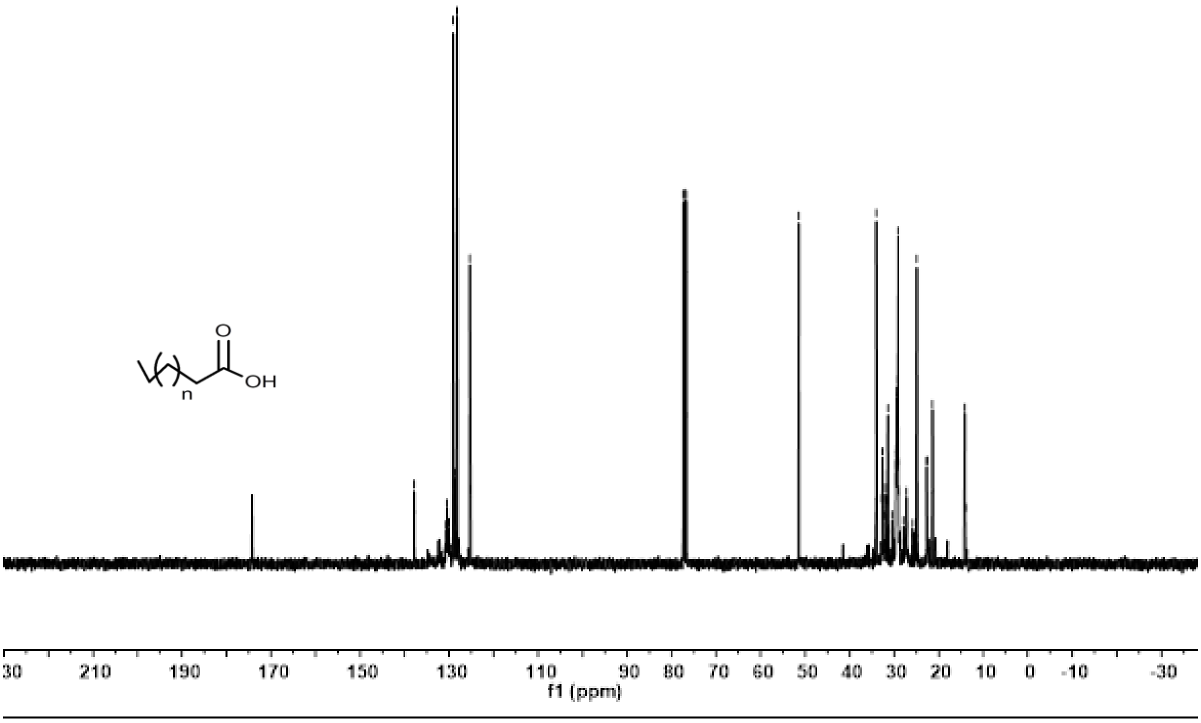

Figure S6 ^{13}C NMR spectrum of isolated product in $CDCl_3$ (after removal of the solvent $MeCN/H_2O$)

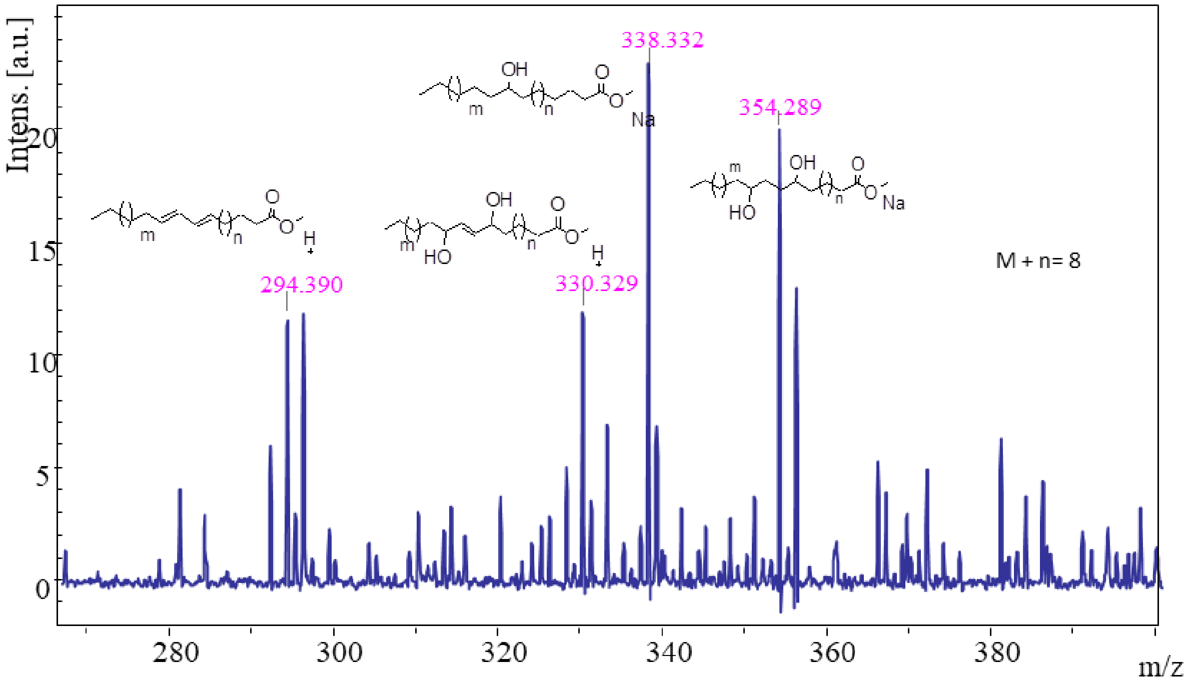

Figure S7. MALDITOF –mass spectroscopy employed for determining the mixture of conjugated hydroxy methyl linoleate CHML after reaction time 24 h
